# Between-class variation and compositional correlates of physical fitness, and the concordance of composite scores with BMI-defined abnormality: a multilevel analysis of annual surveillance records from one Chinese university

**DOI:** 10.3389/fpubh.2026.1896650

**Published:** 2026-09-07

**Authors:** Yan Bu, Junhui Zhu, Mingling Zhao, Ling Xu, Zhongming Song

**Affiliations:** 1 College of Physical Education and Health Science, Yibin University, Yibin, Sichuan, China; 2 Department of Marine Sports, Pukyong National University, Busan, Republic of Korea; 3 Faculty of Education, Universiti Kebangsaan Malaysia, Bangi, Selangor, Malaysia; 4 School of Urban Construction, Chengdu Polytechnic, Chengdu, Sichuan, China; 5 Teaching Management Center, Sichuan Tianfu New Area No. 5 Primary School, Chengdu, Sichuan, China; 6 Physical Education Teaching and Research Office, Tianfu No. 7 Middle School, Chengdu, Sichuan, China

**Keywords:** between-class variation, body mass index, class context, compositional effects, multilevel modelling, physical fitness, surveillance, university students

## Abstract

**Introduction:**

This repeated cross-sectional retrospective study examined between-class variation in university students’ physical-fitness scores and whether annual composite-score trends were concordant with changes in BMI-defined abnormality.

**Methods:**

Annual surveillance records collected from 2020 to 2024 at one public university in Sichuan Province, China, were analysed. The dataset included 106,461 records from 2,591 class-year units (44,240 male, 62,221 female; mean age 20.67 ± 1.54 years; mean BMI 21.64 ± 3.52 kg/m^2^). Multilevel models quantified between-class variation and examined individual- and class-compositional correlates, with calendar year entered categorically. A complementary temporal analysis compared annual composite scores with the prevalence of underweight, overweight, or obesity using Chinese national cut-points.

**Results:**

The null-model intraclass correlation coefficient was 0.178, indicating meaningful between-class clustering. After adjustment, higher within-class BMI, older age, and male sex were associated with lower composite scores. At the class level, higher mean BMI was associated with lower scores, whereas the class-size association was statistically significant but practically negligible. The within-class BMI–fitness association varied across class-year units, although heterogeneity was modest and the association was negative in almost all units. Composite scores increased in 2021, declined in 2022, and increased again in 2023–2024. In contrast, BMI-defined abnormality rose every year, from 23.3% in 2020 to 31.4% in 2024.

**Discussion:**

University fitness data have a meaningful clustered structure, and improvement in a weighted composite score does not necessarily imply parallel improvement in the BMI distribution. Surveillance reports should therefore account for clustering and present composite scores alongside BMI categories and component-specific outcomes.

## 1 Introduction

Physical fitness among university students is an important composite indicator of physical and psychological development in young adults and a key criterion for evaluating health promotion and talent cultivation in higher-education institutions. Nationally, student physical fitness in China continues to face substantial challenges, including fluctuations in endurance, strength, and speed indicators and the coexistence of overweight and obesity with insufficient fitness. Surveillance evidence based on five successive national surveys indicates that overall fitness among Chinese college students changed unfavourably alongside rising cardiovascular risk between 2000 and 2019, suggesting that the university period has become a critical window in which health risks emerge earlier in the life course (1). Because fitness is shaped not only by individual characteristics but also by the settings in which students study and exercise, understanding this problem requires attention to the organizational contexts—such as the class—in which students are embedded (2).

University fitness surveillance commonly summarises multidimensional test performance using a single weighted composite score. Under the National Student Physical Fitness Standards (NSPFS), body mass index (BMI) contributes only 15% of the total, the remainder coming from vital capacity, speed, flexibility, power, and an endurance/strength block. An improvement in the composite score therefore does not necessarily indicate uniform improvement across all underlying health-related indicators: favourable changes in the speed, endurance, or strength components can coexist with an unfavourable shift in the distribution of BMI categories, so that the total score improves while weight status does not. The same logic applies across the settings in which students are nested—a single institution-wide or pooled number can obscure substantial variation between classes. These two forms of hidden heterogeneity—across component signals over time, and across classes at a point in time—motivate the present study. We treat the composite fitness score and BMI-defined abnormality as partially overlapping but non-interchangeable surveillance measures rather than as completely independent constructs, because BMI is itself one weighted component of the total score.

Substantial evidence exists on individual-level determinants of student fitness. The BMI–fitness association is consistently inverse and often non-linear: cross-sectional and multicentre studies of Chinese university students report that both underweight and, especially, overweight and obesity are associated with poorer performance on vital capacity, speed, endurance, and overall fitness, and that even normal-weight obesity is linked to lower fitness (3–5). Sex and academic grade are likewise closely related to fitness levels. However, most studies of university-student fitness treat the individual student as the sole unit of analysis—relying on ordinary regression, between-group comparisons, or descriptive trends [e.g., (3, 4)]—and pay limited attention to the hierarchical structure in which students are nested within classes, majors, and colleges. Fewer studies still ask whether the two most commonly reported surveillance signals, the composite score and the BMI distribution, move together across successive years. The comparatively few multilevel studies in Chinese educational settings (6, 7) illustrate the value of modelling this structure explicitly.

From an educational-organization perspective, the class is not merely a statistical “background variable” but a micro-institutional context in which students’ daily learning, sport participation, peer interaction, and behavioural norms are formed and reproduced. This proximal setting is theoretically relevant to health psychology: the class environment may shape health behaviour through peer norms and shared routines, through a collectively experienced motivational climate (8), and through collective efficacy—a group’s shared belief in its capacity to organize and execute action (9, 10). Under the social-ecological model, individual behaviour and health are jointly shaped by individual, interpersonal, organizational, and institutional levels (2), so that proximal social contexts such as the class can contribute meaningfully to individual outcomes. Consistent with this view, Novak et al. (11) reported substantial school-level variation in youth fitness that remained after adjustment for individual covariates such as sex and class, and research on Chinese school physical-education contexts shows that class-level factors such as class size, lesson location, and lesson content influence the moderate-to-vigorous activity students accumulate (7); cross-level associations among technology use, engagement, and fitness have likewise been observed in university physical education (6).

We emphasise at the outset that the class-level indicators available in the present surveillance database (class size, proportion of male students, mean class BMI) capture class composition rather than directly measured organizational or psychosocial context, and that BMI-defined abnormality is a population-level anthropometric screening indicator rather than a measure of overall health. The study is therefore exploratory and hypothesis-generating. Against this background, the study had four specific aims: (1) to quantify between-class variation in total physical-fitness scores (the ICC); (2) to examine whether class size, the proportion of male students, and mean class BMI are associated with fitness after adjustment for individual characteristics and calendar year; (3) to test whether the within-class BMI–fitness slope varies across classes; and (4) to examine whether annual changes in the composite fitness score and in BMI-defined abnormality were concordant or discordant across 2020–2024. The aim was to characterise the synchrony of two annual surveillance signals—not to establish that fitness and BMI are independent, nor to infer within-student change from repeated cross-sections.

## 2 Materials and methods

### 2.1 Data source and study population

This was a retrospective observational analysis of annual physical-fitness surveillance records collected from 2020 to 2024 at one public university in Sichuan Province, Southwest China. The source database contained grade code, class code, class name, a within-year student identifier, sex, date of birth, height, weight, and, for each fitness component, the raw result, standardized score, and grade, together with bonus points and the total physical-fitness score. All testing followed the National Student Physical Fitness Standards (NSPFS). Under the NSPFS, the total score (0–120, with a bonus component; higher = better) is a weighted composite of body mass index (15%), vital capacity (15%), a 50-m sprint (20%), sit-and-reach flexibility (10%), standing long jump (10%), and an endurance/strength block (30%; the 800-m run and one-minute sit-ups for women, the 1,000-m run and pull-ups for men), with sex- and, where applicable, grade-specific scoring norms. Because scoring norms are sex-specific, the total score is already partly adjusted for sex.

The source surveillance files contained administrative student identifiers. Before statistical modelling, direct identifiers were removed or transformed, and the analytic dataset retained only the variables required for analysis; stable cross-year identifiers were not available to the research team. The de-identified records were made available for research on 16 April 2026. Because a stable cross-year identifier that could deterministically link the same student across the 2020–2024 window was not available, and because identifier-assignment rules differed across cohorts and academic years, longitudinal linkage of individual students across years was not possible. Obtaining stable personal identifiers would exceed the scope of the approved ethics protocol and the institutional data-protection agreement, and re-identification was therefore neither permitted nor feasible. Consequently, the annual record was adopted as the analytic unit; the design is repeated cross-sectional at the record level, within-student correlation across years could not be modelled directly, and calendar-year and grade contrasts are between-pool comparisons rather than within-student change (Sections 2.4 and 4). The class-level unit was defined as a class-year unit—a specific administrative class observed in a specific survey year—because the student membership of a given administrative class changes across years as cohorts enter and leave.

**Figure 1:**
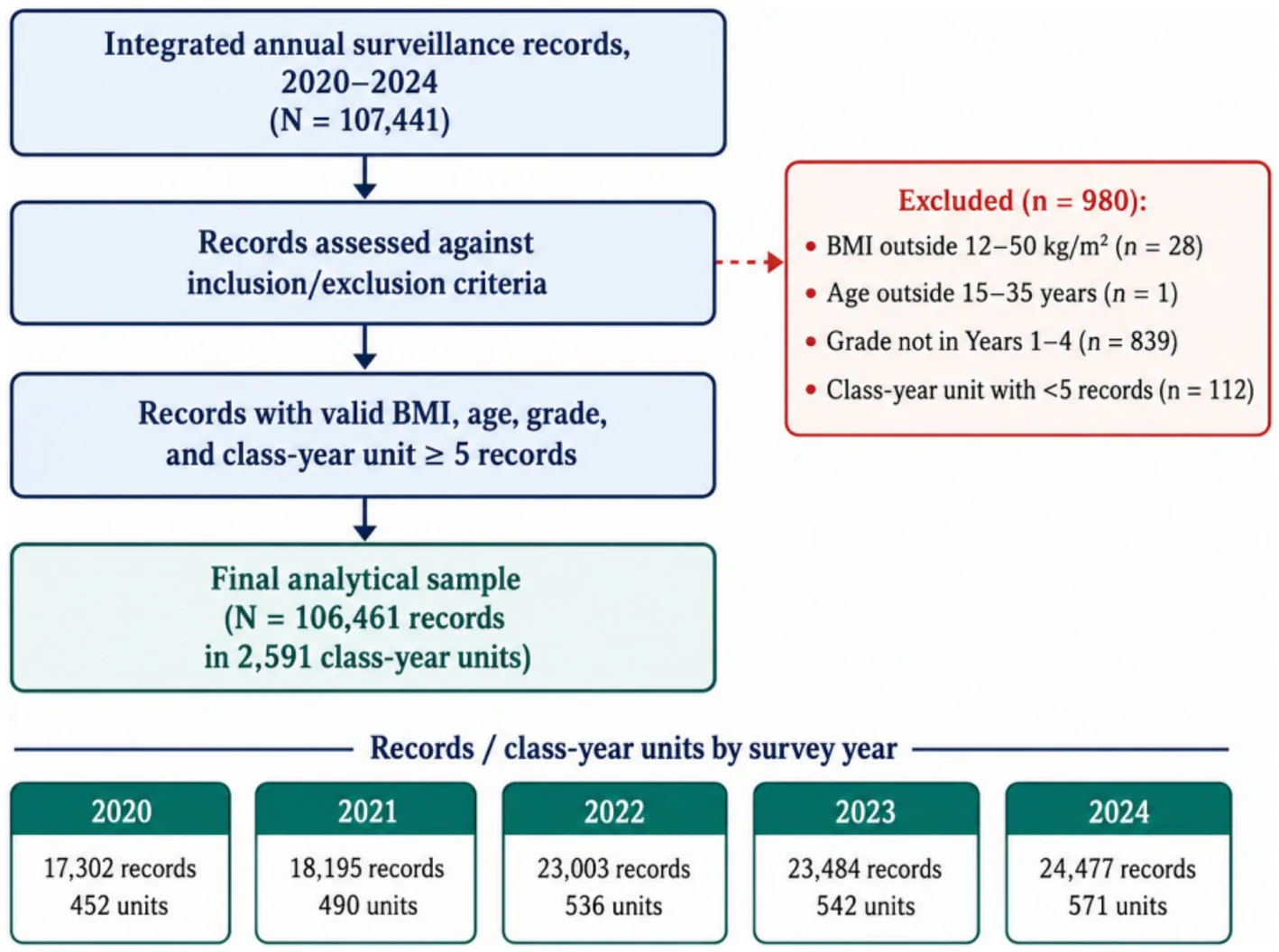
Flow of records through data integration and cleaning (107,441 → 106,461 records analysed in 2,591 class-year units), with the by-year breakdown of the final sample.

After integration, 107,441 records were obtained. Records were excluded for a BMI outside the prespecified 12–50 kg/m^2^ screening range (*n* = 28), an age outside the prespecified 15–35-year range (*n* = 1), unidentifiable academic grade (*n* = 839), and membership in a class-year unit with fewer than five eligible records (*n* = 112), leaving 106,461 eligible records from 2,591 class-year units (Figure 1). A prespecified BMI range of 12–50 kg/m^2^ was used as a data-quality screen for this routine young-adult surveillance dataset; values outside this range were treated as extreme observations likely to reflect measurement or data-entry error. Because rare genuine extreme values cannot be completely excluded, this screening rule is acknowledged as a limitation. Similarly, records with an age outside the prespecified 15–35-year range were treated as likely date-of-birth entry errors. In the retained sample the median class-year size was 41 (range 5–84). Height, weight, BMI, BMI category, and total scores were complete in the retained sample, and no other analysis-relevant variables had missing values.

### 2.2 Variable definitions

*Dependent variable*. The NSPFS total physical-fitness score, treated as continuous.

*Student-level variables*. Sex, BMI, age, and academic grade were level-1 predictors. Age was computed in years from date of birth to 30 September of the survey year. Academic grade (Year 1–4) was derived from the entry year encoded in the student identifier and the survey year (grade = survey year − entry year + 1). BMI was computed as weight (kg) divided by height (m) squared.

BMI category (anthropometric screening classification). In addition to continuous BMI, each record carried the NSPFS weight-status category. BMI categories were read directly from the surveillance database and correspond to the sex-specific cut-points of the National Student Physical Fitness Standards (12), harmonised with the Working Group on Obesity in China criteria (13). Underweight was defined as BMI < 17.2 kg/m^2^ for female students and BMI < 17.9 kg/m^2^ for male students; normal weight as the interval up to but not including 24 kg/m^2^; overweight as 24 ≤ BMI < 28 kg/m^2^; and obesity as BMI ≥ 28 kg/m^2^. The identical classification algorithm was applied to all five survey years. For the temporal analysis (Section 2.7), “BMI-defined abnormality” denotes any category other than normal weight, i.e., the combined prevalence of underweight, overweight, and obesity; the underweight and overweight/obese tails are also reported separately. BMI categories were used as anthropometric screening classifications rather than clinical diagnoses. They were intended to characterise the annual distribution of weight status at the population level and were not interpreted as comprehensive measures of individual health.

Class-level variables. Three level-2 variables were computed within each class-year unit: class size, proportion of male students, and mean class BMI. Calendar year (2020–2024) was entered as a record-level categorical fixed-effect set (dummy coded, 2020 reference).

### 2.3 Centring and interpretation

Individual BMI was centred around the mean BMI of its class-year unit (within-class centring), and the class mean BMI was entered as a separate level-2 predictor, so that within-class and between-class BMI associations could be separated—an approach recommended when contextual and individual associations may differ (14). The within-class BMI coefficient represents the association between a student’s deviation from the class-year mean BMI and that student’s fitness score; the class-mean BMI coefficient represents the association between a unit’s average BMI and fitness scores after adjustment for individual deviation. Age, class size, proportion of male students, and mean class BMI were grand-mean centred so that the intercept refers to an average record in an average class-year unit; sex was coded male = 1, female = 0, and grade and calendar year were dummy coded.

### 2.4 Statistical analysis

Descriptive statistics were computed (mean ± SD for continuous variables; counts and proportions for categorical variables), and included versus excluded records were compared. Analyses used Python 3.12 with statsmodels 0.14.6 (MixedLM); all models were fitted by maximum likelihood (ML) to permit direct AIC/BIC and likelihood-ratio comparison across nested models. A null random-intercept model estimated the between-class variance and the ICC:

Level 1: Y_ij_ = β_0j_ + r_ij_; Level 2: β_0j_ = γ_00_ + u_0j_; ICC = τ_00_/ (τ_00_ + σ^2^), where Y_ij_ is the total score for record i in unit j, r_ij_ the level-1 residual, u_0j_ the class-level random intercept, τ_00_ the between-class variance, and σ^2^ the residual variance.

Three nested specifications were compared: Model 1 (random intercept with individual- and class-level covariates, no calendar-year adjustment); Model 2 (Model 1 plus calendar-year fixed effects); and Model 3, which extended Model 2 by allowing the within-class BMI slope to vary across class-year units. Calendar year was modelled categorically rather than as a linear trend because the 2020–2024 window spanned pandemic disruption (2020–2022), return-to-campus transitions, and possible changes in test administration and scoring (2023–2024); a linear term would impose a monotonic assumption inconsistent with these non-monotonic shifts. The non-significant intercept–slope covariance in Model 3 does not justify discarding the random slope. Model 2 was therefore retained as the primary fixed-effect summary because it corresponds directly to the prespecified individual- and class-level association aims, whereas the better-fitting random-slope specification (Model 3) was used to evaluate whether the within-class BMI association varied across class-year units; the substantive fixed-effect conclusions were unchanged across specifications (Section 3.6; Supplementary Table S7).

Fixed-effect coefficients, standard errors, 95% confidence intervals, standardized coefficients, variance components, AIC, BIC, log-likelihood, and marginal and conditional R^2^ (15) were reported. Nested-model comparisons used likelihood-ratio (LR) tests. Model diagnostics comprised variance inflation factors (VIFs) for all fixed effects, residual-versus-fitted and normal Q–Q plots, and a cluster-level influence analysis in which class-year units with standardized random intercepts beyond ±3 SD were flagged and the model refitted after their removal.

*What the clustering and single-year analyses can and cannot address*. The class-year random intercept accounts for correlation among records from students tested within the same administrative class and survey year; it does not account for repeated observations of the same student across years. Because stable cross-year identifiers were unavailable and re-identification was neither permitted nor feasible, individual longitudinal trajectories could not be reconstructed, and student fixed-effects models—which require the same individual to be identified in more than 1 year—could not be estimated (technically because no reliable cross-year key exists, and ethically because deriving one would breach the data-protection agreement). To partly compensate, the model was refitted separately within each survey year (each student contributing at most one record), calendar year was entered as a fixed-effect set, and single-year sensitivity analyses were reported. These devices reduce, but do not remove, the confounding of period, cohort-composition, and administration effects; they do not license within-student interpretation, and we use the term repeated cross-sectional analysis throughout.

### 2.5 Ethics statement

This study was approved by the Institutional Review Board of the Science and Technology Ethics Committee of Yibin University (approval No. YBULLXS26041601). The requirement for written informed consent was waived by the Institutional Review Board because the analysis used de-identified routine surveillance records and posed no more than minimal risk to participants. All procedures were conducted in accordance with the Declaration of Helsinki and the relevant national and institutional requirements for the secondary analysis of de-identified administrative data. Yibin University was the approving body as the institution responsible for the data.

### 2.6 Test administration and cross-year comparability

All five annual rounds followed the same NSPFS framework and the same sex- and grade-specific scoring algorithm, testing was conducted at the same institution, and the survey files were compiled in comparable autumn windows each year. The surveillance database did not, however, retain complete round-by-round records of equipment model and calibration, examiner assignment and training, ambient testing conditions, make-up-test arrangements, or student effort and motivation; where such information was unavailable this is stated rather than assumed. This limitation is material because calendar-year fixed effects adjust for average differences between survey years but cannot correct measurement non-equivalence caused by changes in equipment, administration, examiner behaviour, or student effort. Annual differences should therefore be interpreted as differences in recorded surveillance outcomes rather than as pure biological changes in fitness, and this concern applies to all test components—not only vital capacity.

### 2.7 Temporal concordance analysis and data-quality checks

To address whether the composite fitness score and BMI-defined abnormality moved concordantly across the window (aim 4), we computed, for each survey year, the mean composite score and the prevalence of BMI-defined abnormality, with 95% confidence intervals and both adjacent-year and 2020-referenced contrasts. Adjusted composite-score estimates were obtained from linear models including age, sex, and academic grade with cluster-robust standard errors (clustered on the class-year unit). Annual BMI-defined abnormality prevalence was directly standardised to the pooled sex and grade distribution; logistic models additionally estimated adjusted odds ratios for each year relative to 2020. An adjacent-year interval was classified as concordant when the two indicators moved in the same prespecified surveillance direction (a higher composite score together with a lower abnormality prevalence, or a lower composite score together with a higher abnormality prevalence) and discordant otherwise. This classification describes directional agreement between surveillance indicators and does not imply equivalent changes in overall health. Because BMI-defined abnormality is a distributional endpoint that could in principle be sensitive to measurement or entry artefacts, each year’s height, weight, and BMI records were screened for implausible values, terminal-digit heaping (assessed as the proportion of integer-ending height and weight values), exact duplicate records, and constant-value (single-height or single-weight) classes; the 2024 increase—the largest and most recent—was further examined by stratifying across sexes, grades, and classes and by repeating the comparison after excluding very-low-weight records. Full quality-check results are provided in Supplementary Tables S4–S5 and Supplementary Figure S2.

## 3 Results

### 3.1 Sample characteristics

The analytic sample comprised 106,461 records from 2,591 class-year units. Mean age was 20.67 ± 1.54 years and mean BMI 21.64 ± 3.52 kg/m^2^; 44,240 records (41.6%) were from male and 62,221 (58.4%) from female students. The mean total physical-fitness score was 71.60 ± 8.56. Records from male students had higher mean BMI (22.39 vs. 21.11 kg/m^2^) and lower mean composite scores (68.80 vs. 73.59) than records from female students. Because the component tests and scoring norms are sex-specific, this difference should be interpreted as a contrast in the resulting composite score rather than as a direct measure of underlying biological fitness. The median class-year size was 41 (mean 41.09 ± 12.63; range 5–84); the mean within-unit proportion of male students was 0.406 ± 0.257 and the mean class BMI 21.63 ± 0.81 kg/m^2^. Sample sizes were broadly comparable across survey years, ranging from 17,302 records (452 units) in 2020 to 24,477 records (571 units) in 2024 (Table 1).

**Table 1:** Sample characteristics overall and by sex, and annual surveillance summary, 2020–2024.

Panel A. Record-level characteristics overall and by sex			
Characteristic	Overall	Male	Female
Records, *n*	106,461	44,240	62,221
Age, years	20.67 ± 1.54	20.79 ± 1.55	20.59 ± 1.53
BMI, kg/m^2^	21.64 ± 3.52	22.39 ± 3.86	21.11 ± 3.17
Total physical-fitness score	71.60 ± 8.56	68.80 ± 8.72	73.59 ± 7.86

Panel B. Surveillance records and class-year units by survey year				
Survey year	Records, *n*	Class-year units, *n*	Mean class-year size	Mean total score
2020	17,302	452	38.3	70.72
2021	18,195	490	37.1	73.37
2022	23,003	536	42.9	69.86
2023	23,484	542	43.3	71.00
2024	24,477	571	42.9	73.11

Continuous variables are presented as mean ± SD unless otherwise indicated. The analytic sample comprised 106,461 records nested within 2,591 class-year units. The overall mean class-year size was 41.09 ± 12.63 records (median 41, range 5–84), the mean proportion of male students was 0.406 ± 0.257, and the mean class BMI was 21.63 ± 0.81 kg/m^2^.

**Figure 2:**
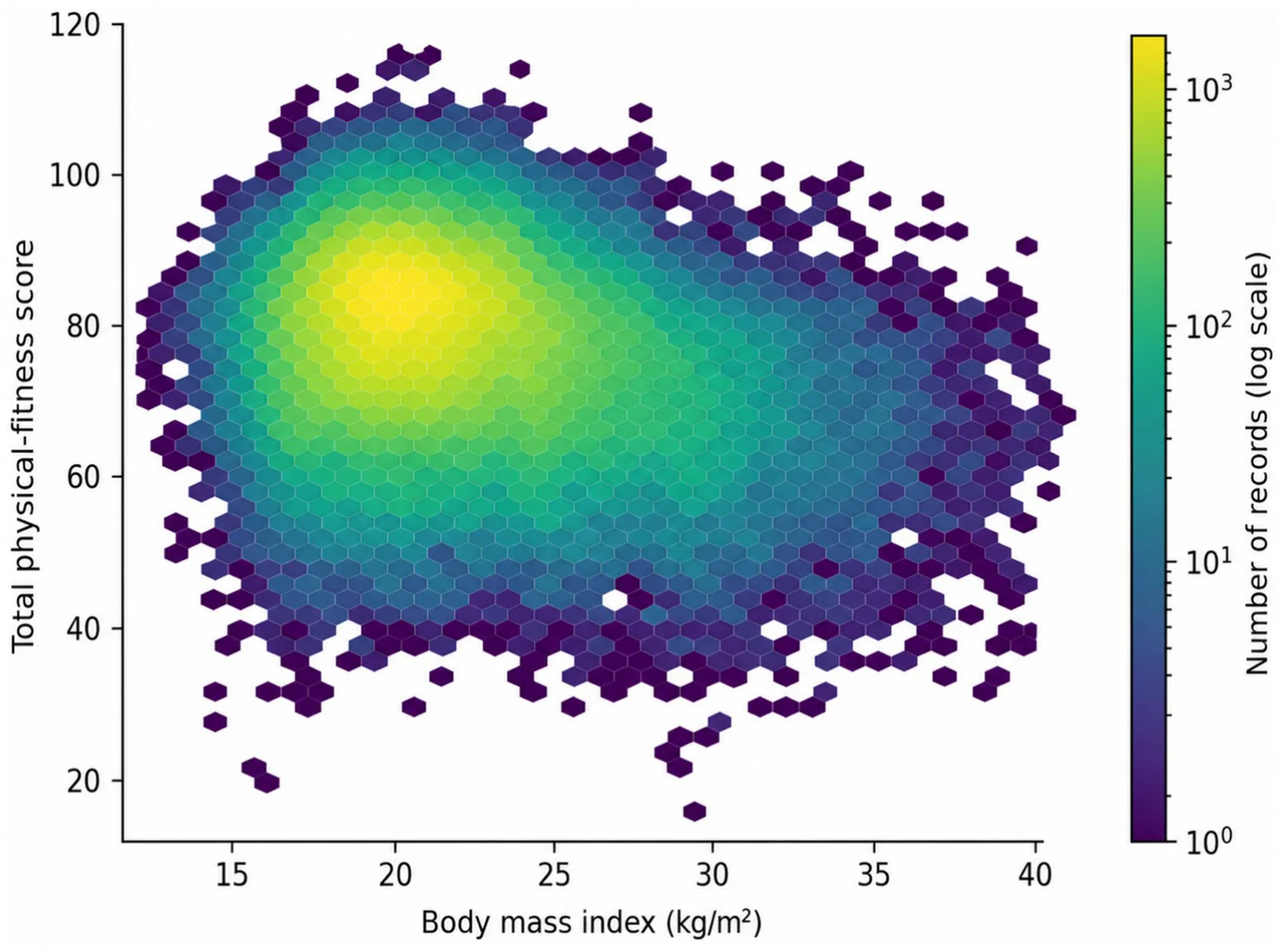
Hexbin density of individual BMI versus total physical fitness score across all 106,461 records.

The overall joint distribution of individual BMI and total physical-fitness score across the analytic sample is shown in Figure 2.

### 3.2 Included versus excluded records

A total of 980 records were excluded during data cleaning. Compared with included records, excluded records were on average older (22.71 vs. 20.67 years, *p* < 0.001) and had higher BMI (22.93 vs. 21.64 kg/m^2^, *p* < 0.001) and a higher proportion of male students (68.1% vs. 41.6%, *χ*^2^ *p* < 0.001), reflecting that most exclusions were fifth-year or repeat students whose grade could not be validly assigned. The mean composite score was nearly identical in included and excluded records (71.60 vs. 71.62; *t*-test *p* = 0.946). Thus, exclusion did not materially alter the unadjusted mean outcome, although bias in covariate distributions or in adjusted associations cannot be ruled out (Table 2).

**Table 2:** Comparison of included and excluded records.

Variable	Included (*n* = 106,461)	Excluded (*n* = 980)	*p*
Age, years	20.67 ± 1.54	22.71 ± 1.57	<0.001
BMI, kg/m^2^	21.64 ± 3.52	22.93 ± 6.88	<0.001
Male, %	41.6	68.1	<0.001
Total score	71.60 ± 8.56	71.62 ± 8.40	0.946

Marginal *R*^2^ = 0.154; conditional *R*^2^ = 0.282.

### 3.3 Between-class variation (null model)

The null random-intercept model yielded a between-class variance of 13.08 and a residual variance of 60.23, giving an ICC of 0.178. Thus roughly 18% of the total variance in individual fitness scores lay between class-year units, indicating substantial clustering and confirming that a multilevel approach was warranted. Between-class variation was present in every survey year when models were fitted separately (null-model ICC 0.097–0.253; Section 3.7 and Supplementary Table S1), showing that the clustering is not an artefact of pooling across years.

### 3.4 Fixed-effect associations (model 2, primary specification)

In the primary specification (Model 2), higher within-class BMI was associated with lower fitness (*B* = −0.528, 95% CI − 0.540 to −0.515, *p* < 0.001), as were older age (*B* = −0.128, *p* < 0.001) and male sex (*B* = −4.174, 95% CI − 4.280 to −4.068, *p* < 0.001). Relative to records from first-year students, records from second-year (*B* = 0.803, *p* < 0.001) and fourth-year students (*B* = 1.913, *p* < 0.001) had higher adjusted scores, whereas third-year records did not differ significantly (*B* = 0.283, *p* = 0.078); these are cross-sectional contrasts between grade-specific record pools rather than within-student change (Section 4.3). At the class level, a higher proportion of male students was associated with higher fitness (*B* = 1.090, 95% CI 0.557 to 1.622, *p* < 0.001), while larger class size (*B* = −0.012, *p* = 0.021) and higher mean class BMI (*B* = −0.906, 95% CI − 1.080 to −0.731, *p* < 0.001) were associated with lower fitness. Calendar-year effects were non-monotonic, with 2024 (*B* = 3.057) and 2021 (*B* = 2.805) highest relative to 2020 and 2022 slightly lower (*B* = −0.372, *p* = 0.079), supporting categorical rather than linear modelling of year. Standardized coefficients (*β*) indicated that sex (*β* = −0.240) and within-class BMI (*β* = −0.212) were the strongest individual-level correlates; mean class BMI was the largest class-level correlate (*β* = −0.084). The fixed effects accounted for 15.4% of the variance (marginal R^2^ = 0.154) and the full model 28.2% (conditional R^2^ = 0.282). Full estimates are given in Table 3.

**Table 3:** Model 2 (primary specification) fixed effects: Unstandardized coefficients (*B*), 95% CIs, standardized coefficients (β*), and *p*-values. Reference categories: Female (sex), Year 1 (grade), 2020 (survey year).

Predictor	*B*	95% CI	β*	*p*
Intercept	71.291	70.922, 71.661	—	<0.001
Grade 2 (vs 1)	0.803	0.494, 1.111	0.042	<0.001
Grade 3 (vs 1)	0.283	−0.032, 0.598	0.014	0.078
Grade 4 (vs 1)	1.913	1.577, 2.250	0.092	<0.001
Sex (male vs. female)	−4.174	−4.280, −4.068	−0.240	<0.001
BMI (within-class)	−0.528	−0.540, −0.515	−0.212	<0.001
Age (centred)	−0.128	−0.172, −0.085	−0.023	<0.001
Class size (centred)	−0.012	−0.023, −0.002	−0.017	0.021
Prop. male (centred)	1.090	0.557, 1.622	0.033	<0.001
Mean class BMI (centred)	−0.906	−1.080, −0.731	−0.084	<0.001
Year 2021 (vs 2020)	2.805	2.379, 3.230	0.123	<0.001
Year 2022 (vs 2020)	−0.372	−0.787, 0.043	−0.018	0.079
Year 2023 (vs 2020)	1.062	0.632, 1.492	0.051	<0.001
Year 2024 (vs 2020)	3.057	2.642, 3.472	0.150	<0.001

**Figure 3:**
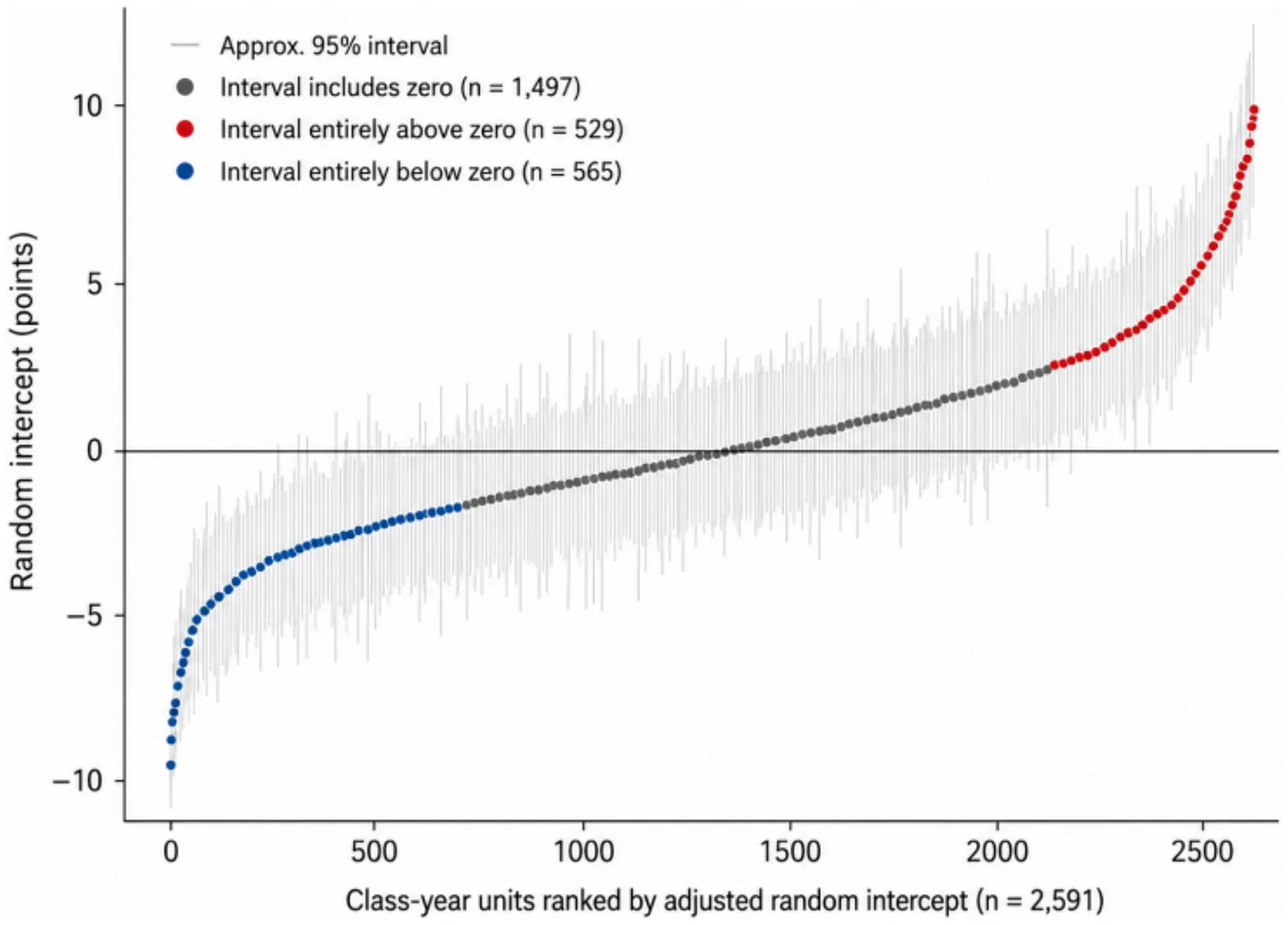
Caterpillar plot of class-year random intercepts (Model 2, empirical-Bayes estimates with approximate 95% intervals). Intervals entirely above zero (*n* = 529) and entirely below zero (*n* = 565) are distinguished from intervals that include zero (*n* = 1,497). These intervals are descriptive summaries of the estimated random effects and are not multiple-comparison-corrected significance tests.

Beyond the fixed effects, the adjusted class-year random intercepts from Model 2 spanned roughly 20 points (Figure 3): of the 2,591 class-year units, 529 had empirical-Bayes 95% intervals entirely above zero, 565 had intervals entirely below zero, and 1,497 had intervals that included zero.

### 3.5 Model comparison

Adding individual- and class-level covariates (Model 1) improved fit markedly over the null model (LR *χ*^2^ = 14,656.80, df = 9, *p* < 0.001). Adding calendar-year fixed effects (Model 2) produced a further significant improvement (LR *χ*^2^ = 435.04, df = 4, *p* < 0.001) and was adopted as the primary specification. Adding a random slope for within-class BMI (Model 3) again improved fit (LR *χ*^2^ = 463.07, df = 2, *p* < 0.001). The adjusted ICC from Model 2 was 0.151, showing that individual and class composition explained part—but by no means all—of the between-class variation observed in the null model (Table 4).

**Table 4:** Model comparison: All models fitted by maximum likelihood.

Model	ICC	AIC	BIC	LR test vs previous
Null (random intercept)	0.178	744,259	744,288	—
Model 1 (+ covariates)	0.177	729,621	729,735	*χ*^2^ = 14,656.80, df = 9***
Model 2 (+ year)	0.151	729,193	729,347	*χ*^2^ = 435.04, df = 4***
Model 3 (+ random slope)	—	728,734	728,907	*χ*^2^ = 463.07, df = 2***

***p < 0.001.

### 3.6 Random-slope heterogeneity and covariance test

**Figure 4:**
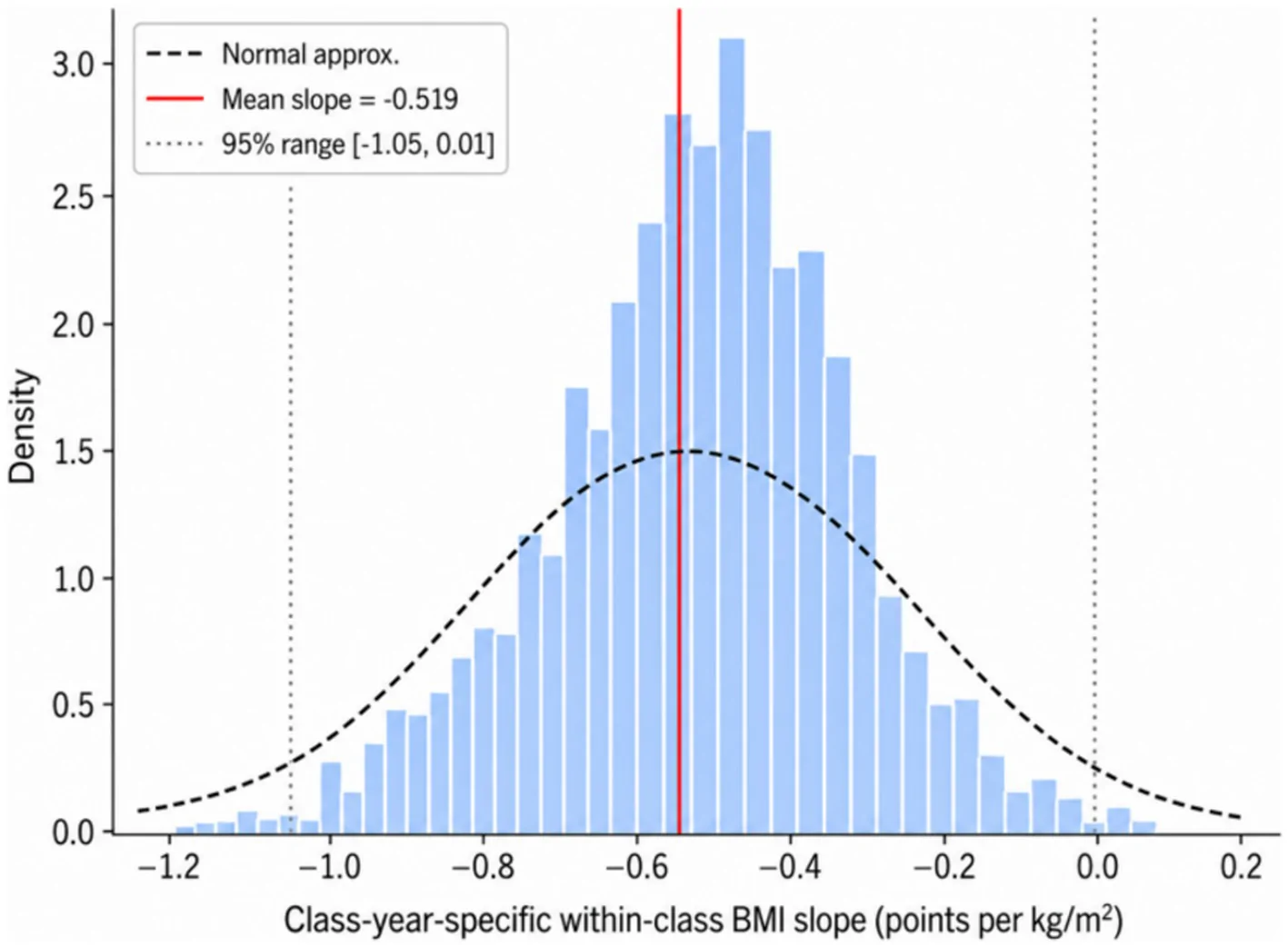
Distribution of class-specific within-class BMI slopes (Model 3, empirical-Bayes estimates). The mean slope is −0.52 and ≈95% of slopes fall between −1.05 and +0.01; fewer than 4% are positive.

In Model 3 the within-class BMI slope varied across class-year units, with an estimated slope variance of 0.073 (SD ≈ 0.27). The mean slope was −0.519, and approximately 95% of class-specific slopes fell between −1.05 and +0.01; fewer than 4% of units had a positive empirical-Bayes slope, so the negative within-class BMI–fitness association held in almost all classes while varying in magnitude (Figure 4). Although this heterogeneity was statistically significant, its practical scale was modest. The covariance between the random intercept and the random BMI slope was small and not statistically significant (covariance = 0.039; correlatio*n* = 0.048); a non-significant covariance argues only against retaining the intercept–slope correlation, not against the random slope itself. Because the substantive fixed-effect estimates were essentially identical under the random-intercept and random-slope specifications (Supplementary Table S7), Model 2 was retained as the primary fixed-effect summary, as it corresponds directly to the prespecified individual- and class-level association aims, whereas the better-fitting random-slope Model 3 was used to characterise the heterogeneity in the within-class BMI association across class-year units. The substantive fixed-effect conclusions were unchanged across the two specifications.

### 3.7 Diagnostics and sensitivity analyses

Variance inflation factors for all Model 2 fixed effects were low (all < 2.1), indicating no problematic multicollinearity. Residual-versus-fitted plots showed no marked heteroscedasticity, and the normal Q–Q plot indicated approximately normal residuals with mild left skew (−0.48) that is inconsequential at this sample size (Supplementary Figure S1). A cluster-level influence analysis identified 14 class-year units with standardized random intercepts beyond ±3 SD; refitting Model 2 after removing these units left all key coefficients essentially unchanged (e.g., mean class BMI − 0.906 → −0.934; sex −4.174 → −4.150; within-class BMI − 0.528 → −0.529).

Because individual students could not be linked across years, the model was refitted separately within each survey year. Substantial between-class variation was present in all 5 years (null-model ICC 0.097–0.253), and the direction of the key associations was stable: within-class BMI, mean class BMI, and male sex were negative and significant in every year, while the proportion of male students was positive and significant in 2022 and 2023 and non-significant in 2020, 2021, and 2024 (Table 5; selected single-year estimates in Supplementary Table S1). The magnitude of the sex coefficient varied across years (from −0.45 in 2020 to −9.00 in 2022), mirroring a genuine year-to-year fluctuation in the raw male–female score gap and reinforcing the decision to model calendar year categorically. Additional models that added mean class age as a level-2 predictor, or that removed age or grade, left the class-level associations robust (Supplementary Table S3).

**Table 5:** Single-year sensitivity analysis: between-class ICC and key coefficients by survey year (each student contributes at most one record).

Year	ICC null	ICC adj	BMI (w/in)	Sex	Prop male	Mean BMI
2020	0.097	0.093	−0.455***	−0.448***	−0.890	−0.451*
2021	0.132	0.084	−0.526***	−4.771***	0.096	−0.553***
2022	0.253	0.168	−0.538***	−9.003***	2.718***	−1.718***
2023	0.129	0.092	−0.644***	−4.332***	2.027***	−1.726***
2024	0.117	0.080	−0.461***	−1.348***	0.732	−0.291*

*p < 0.05; ***p < 0.001. “Prop male” and “Mean BMI” are class-level (level-2) coefficients; “BMI (w/in)” is the within-class individual coefficient.

### 3.8 Concordance of the composite score with BMI-defined abnormality (2020–2024)

**Figure 5:**
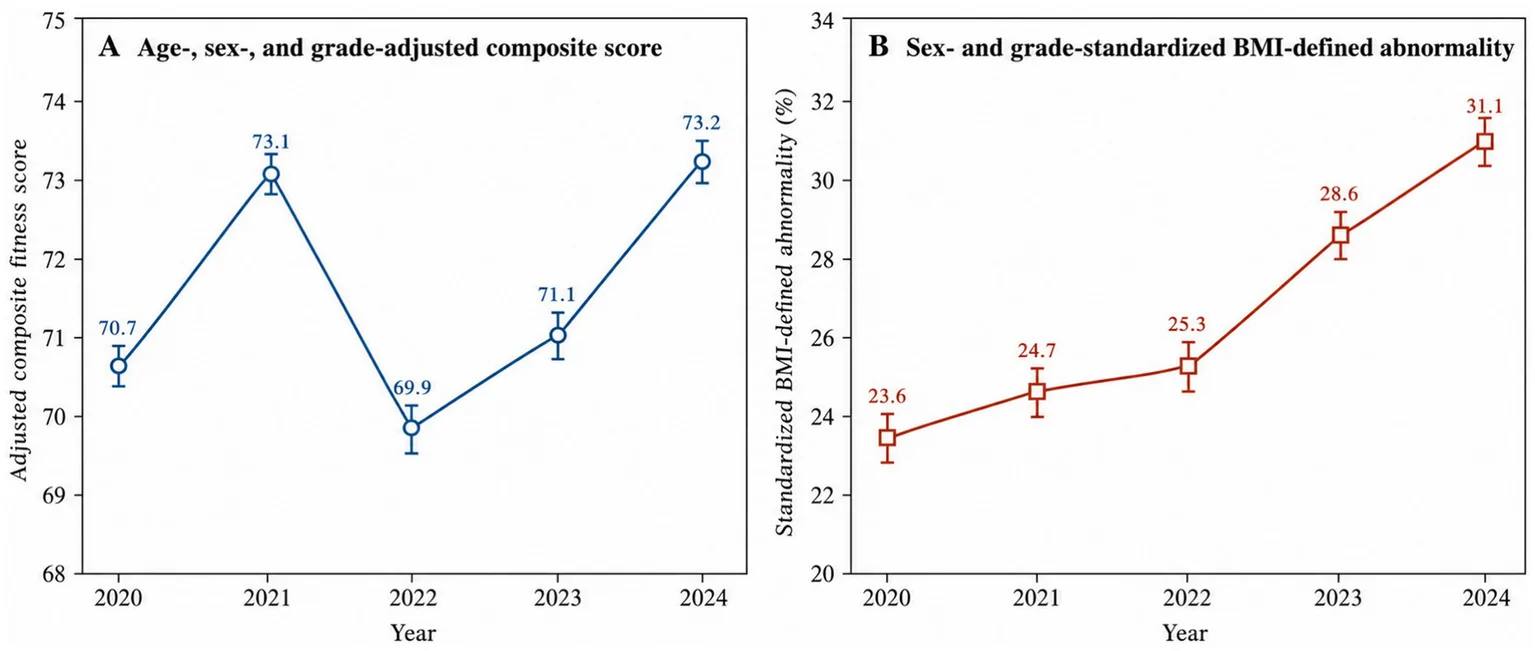
Annual trajectories of the composite fitness score and BMI-defined abnormality, 2020–2024. Panel **(A)** shows age-, sex-, and grade-adjusted composite-score estimates (non-monotonic: An increase in 2021, a decline in 2022, and a further increase in 2023–2024). Panel **(B)** shows BMI-defined abnormality prevalence directly standardised to the pooled sex and grade distribution (a monotonic rise from 23.6 to 31.1%). Error bars are 95% confidence intervals. Separate panels are presented because the indicators are measured on different scales and should not be interpreted as directly interchangeable.

Across the 5 years the composite fitness score followed a non-monotonic path, whereas BMI-defined abnormality rose steadily (Table 6 and Figure 5). The mean composite score increased from 70.72 in 2020 to 73.37 in 2021, declined to 69.86 in 2022, and increased again to 71.00 (2023) and 73.11 (2024); after adjustment for age, sex, and grade the same shape held (adjusted contrasts versus 2020: +2.41, −0.75, +0.38, +2.55 points). BMI-defined abnormality, by contrast, increased in every adjacent-year interval, from 23.32% (2020) to 24.15% (2021), 25.43% (2022), 28.81% (2023) and 31.40% (2024); adjusted year contrasts versus 2020 were +1.39, +1.74, +4.99, and +7.42 percentage points, and adjusted odds ratios were 1.08, 1.10, 1.31, and 1.47 (all *p* ≤ 0.005). Direct sex- and grade-standardisation left the trend essentially unchanged (23.6% → 31.1%), indicating that it was not driven by shifts in the sex or grade composition of the annual pools.

**Table 6:** Annual composite fitness score and BMI-defined abnormality, 2020–2024, with adjusted contrasts versus 2020 and adjacent-year concordance.

Year	Composite score (mean)	Adj. Δ vs 2020	BMI-abnormality (%)	Adj. Δ vs 2020 (pp)	Adjacent-year concordance
2020	70.72	ref	23.32	ref	—
2021	73.37	+2.41	24.15	+1.39	Discordant
2022	69.86	−0.75	25.43	+1.74	Concordant (both worsen)
2023	71.00	+0.38	28.81	+4.99	Discordant
2024	73.11	+2.55	31.40	+7.42	Discordant

Classifying each adjacent-year interval by whether the two indicators moved in the same prespecified surveillance direction, the signals were already discordant in 2020–2021 (composite score up 2.65 points while abnormality rose 0.83 points); they moved concordantly—both worsening—only during the 2021–2022 decline; and they were discordant again in 2022–2023 and 2023–2024, the composite score increasing again while abnormality continued to rise. The divergence was therefore not confined to the post-2022 period: it was present from the start of the window and, apart from the 2022 decline, persisted throughout. The rise in abnormality reflected both tails of the distribution—overweight-plus-obesity increased from 16.9% (2020) to 23.3% (2024), while the underweight fraction, stable near 6% through 2022, rose to 8.1% in 2024.

Because the 2024 abnormality level was the highest observed, the 2024 height, weight, and BMI records were examined for data-quality problems (Supplementary Tables S4–S5). Terminal-digit patterns in 2024 were consistent with the low-heaping years 2020, 2021, and 2023 (about 10% integer endings for both height and weight); the only year showing marked heaping was 2022 (about 28% integer endings), not 2024. This pattern indicates lower recording precision for anthropometric measurements in 2022, although it does not establish that the composite-score decline in 2022 was caused by measurement error, because BMI contributed only part of the total score. No exact duplicate records and no constant-value (single-height or single-weight) classes were found in any year. Very-low-weight records (body weight < 35 kg, treated as a sensitivity-screening threshold rather than an exclusion criterion or evidence of data error) were uncommon in 2020–2023 but more frequent in 2024, when 61 records (0.25% of the year) fell below this threshold, consistent with the larger underweight tail observed in 2024; excluding them left the 2024 abnormality prevalence essentially unchanged (31.40% → 31.23%). The increase was broadly distributed rather than concentrated: among 368 administrative class codes observed in both the 2023 and 2024 surveillance files, 59% had a higher recorded abnormality prevalence in 2024 (mean change +2.9 percentage points), and the increase appeared in both sexes (female 22.5% → 24.9%; male 37.3% → 39.7%) and in every grade. These comparisons do not represent within-student change because class membership may have differed between years. The 2024 increase thus persisted after data-quality screening, standardisation, and sensitivity analyses, and is unlikely to be explained solely by implausible values or a small number of anomalous classes. Consistent with the observational design, we do not attribute it to any single cause; annual differences in cohort composition, post-pandemic lifestyle patterns, and test administration cannot be separated in these data (Section 4).

Adjusted contrasts are from models with age, sex, and grade and cluster-robust standard errors (clustered on the class-year unit). “Concordance” classifies each adjacent-year interval by whether the two indicators moved in the same prespecified surveillance direction (higher score with lower abnormality, or lower score with higher abnormality); it describes directional agreement and does not imply equivalent changes in overall health. Adjusted year contrasts versus 2020 were all significant (*p* ≤ 0.005); 95% confidence intervals are shown in Figure 5 and full estimates in Supplementary Table S6.

## 4 Discussion

### 4.1 Summary of main findings

Using 106,461 annual surveillance records from 2,591 class-year units at one Chinese university, this study documented four main findings. First, between-class variation in physical fitness was substantial: about 18% of the variance in total scores lay between class-year units, and meaningful between-class variation was present in every survey year. Second, after adjustment for individual characteristics and calendar year, both individual and class-compositional factors were associated with fitness—within-class BMI, age, and male sex negatively at the individual level; and, at the class level, mean class BMI and class size negatively and the proportion of male students positively. Third, the within-class BMI–fitness association varied across classes, though the variation was modest in magnitude and almost always negative in direction. Fourth, the composite fitness score and BMI-defined abnormality diverged across 2020–2024: recorded composite scores followed a non-monotonic pattern and returned to a higher recorded level in 2024, whereas BMI-defined abnormality rose in every year to 31.4% in 2024, a discordance already present in 2020–2021 that persisted across the available data-quality checks. These findings are descriptive and compositional; they identify where variation lies and whether two surveillance signals move together, not why either arises.

### 4.2 Between-class variation and its relevance to health psychology and research design

The ICC of 0.178 indicates that the class-year unit is a non-trivial level of clustering in university-student fitness. This is consistent with Novak et al. (11), who reported substantial school-level variation in youth fitness that persisted after adjustment for sex and class, and with evidence that proximal educational settings shape students’ activity and health behaviour (6, 7). From a health-psychology perspective, the class is a plausible carrier of peer norms, motivational climate, and collective efficacy (8–10): students in the same class share schedules, physical-education instructors, facilities, and social comparisons, any of which could generate correlated fitness outcomes. We stress, however, that our data contain no direct measures of these constructs; the between-class variation we document is an empirical target that such mechanisms could later be tested against, not evidence for them. Methodologically, the sizeable ICC also carries a clear implication: single-level analyses of university-student fitness that ignore class clustering will understate standard errors and risk over-stating the precision of individual-level associations.

### 4.3 Individual-level correlates

The negative within-class BMI coefficient reproduces, at the individual level, the well-documented inverse BMI–fitness association in Chinese students (3–5). Because BMI also contributes 15% of the NSPFS total score directly, part of this association is definitional; the remainder reflects the poorer performance of higher-BMI students on endurance, speed, and strength components. Standardized coefficients placed sex and within-class BMI as the two strongest individual-level correlates. The sex coefficient reflects the combined effects of sex-specific test components, scoring norms, component performance, and annual sample composition, and should not be interpreted as a simple biological sex effect; its magnitude varied across years with the raw male–female score gap.

The higher adjusted scores of records from second- and fourth-year students, relative to first-year students, should be read as cross-sectional contrasts between grade-specific record pools, not as evidence of within-student progression. Because the data are repeated cross-sections and individual students could not be linked across years, the Year-3 records in 1 year are not the same students as the Year-3 records in another, and no developmental interpretation—maturation, adaptation to testing, or attrition of less-fit students as they advance—is warranted. The differences may instead reflect cohort composition, selective continuation, or curriculum and testing arrangements that differ between grade groups; the non-monotonic grade pattern is consistent with such compositional explanations rather than with a smooth developmental gradient.

### 4.4 Compositional versus contextual class-level associations

At the class level, higher mean BMI was the strongest compositional correlate of lower fitness, and a higher proportion of male students was associated with higher average scores, while larger classes scored marginally lower. It is essential to interpret these as compositional rather than contextual associations. Mean class BMI is an aggregate of the individual BMIs within the unit, and the proportion of male students summarises its sex composition; both may capture who is in the class rather than any causal property of the class environment. The class-size association, though statistically significant, was practically negligible—about a 0.24-point difference in total score across a 20-student difference in class size—and should not be over-interpreted. Separating within-class from between-class BMI associations through centring (14) clarified that individual deviations from the class mean and the class mean itself were each independently associated with fitness, but it cannot by itself distinguish composition from context.

### 4.5 Concordance between composite fitness and BMI-defined abnormality

The five-year surveillance signals illustrate why a single composite score can mask underlying change. The composite fitness score and BMI-defined abnormality did not move together: the composite score was non-monotonic, whereas abnormality rose in every year and reached its highest level in 2024. Read only from the composite score—which was as high in 2024 as at any point in the window—one might conclude that student fitness had returned to or exceeded its 2020 level; read from the BMI distribution, the picture is one of steadily rising abnormality. This is the concrete surveillance meaning of the composite-masking problem that motivated the study: because BMI contributes only 15% of the weighted total, gains on the speed, endurance, and strength components can offset an unfavourable shift in the BMI distribution, so that the total score improves while weight status does not. That the two signals were already discordant before the 2022 trough argues against interpreting the divergence as a purely post-pandemic artefact of a single year.

Two interpretive cautions apply. First, these are two related but non-equivalent indicators, not independent constructs; because BMI is itself a weighted component of the total, part of any concordance is structural, and we therefore describe the signals as partially overlapping but non-interchangeable rather than as decoupled or independent. Second, BMI-defined abnormality is a population-level anthropometric screening indicator, not a measure of overall health. BMI cannot distinguish fat mass from lean mass, does not capture central adiposity or metabolic status, and may misclassify muscular individuals (16); the rise we report is a change in the recorded distribution of a weight-status classification and should not be read as demonstrating deterioration in students’ overall health. Within these limits, the finding that the two signals diverged from 2020 to 2024 supports reporting and interpreting composite fitness and anthropometric indicators separately rather than relying on the total score alone.

### 4.6 Practical implications

The results support two cautious, directly grounded implications. For analysis, university fitness data should be handled with multilevel or cluster-robust methods, because a substantial share of the variance lies between class-year units and single-level analyses would overstate precision. For surveillance reporting, composite fitness scores should be presented alongside BMI categories and, where possible, component-specific results, rather than relying on the total score alone; where the composite score and the BMI distribution appear to diverge, the discordance should prompt verification of data quality and testing procedures before substantive or policy conclusions are drawn. These findings do not establish the effectiveness of any particular surveillance or management model, and we make no such claim. Where class-level screening is used to allocate physical-education resources, it should be based on directly measured need rather than on sex or BMI composition, to avoid stigmatising groups.

### 4.7 Strengths and limitations

This study has notable strengths: a very large multi-year surveillance sample, detailed documentation of data cleaning, model specification, diagnostics, and sensitivity analyses, and explicit within/between separation of BMI. Several limitations must be acknowledged. First and most important, the design is repeated cross-sectional at the record level: because a stable cross-year identifier was not available, students could not be linked longitudinally, within-student correlation across years could not be modelled, student fixed-effects models were not feasible, and calendar-year and grade contrasts are between-pool comparisons rather than within-student change; the single-year analyses partly mitigate but do not resolve this. Second, the class-level variables are compositional summaries, not measured features of the class environment. Third, no psychosocial, behavioural, lifestyle, or physical-activity variables (e.g., sleep, diet, training, motivation, peer norms) were available, so the fixed effects explain a limited share of variance (marginal *R*^2^ = 0.154) and residual confounding is likely. Fourth, BMI is a limited anthropometric indicator: it cannot distinguish fat from lean mass, does not capture central adiposity or metabolic status, and may misclassify muscular individuals, so we do not infer overall health deterioration from changes in BMI categories alone. Fifth, although all rounds followed the same national framework, complete information on equipment calibration, examiner assignment, testing conditions, make-up testing, and student effort was unavailable; calendar-year fixed effects adjust for average year differences but cannot correct measurement non-equivalence, and this concern applies to every test component, not only vital capacity. Consistent with this, the greater terminal-digit heaping observed in the 2022 height and weight records suggests that anthropometric recording precision may not have been completely equivalent across years; accordingly, the 2022 decline should not be interpreted as a purely biological or behavioural change. Finally, the data come from a single university in one province, so generalisability is limited and calendar-year effects conflate secular, compositional, administrative, and measurement changes.

### 4.8 Future directions

Future work should, first, obtain ethically governed stable identifiers to build genuinely longitudinal multilevel models of within-student fitness trajectories—the only design that could separate the period, cohort, and administration effects that repeated cross-sections confound here; second, measure class-level psychosocial and behavioural constructs directly—motivational climate, peer norms, collective efficacy, instructor characteristics, and facility access—to test whether they account for the between-class variation documented here; third, extend the analysis to multiple institutions and provinces and add higher levels (major, college, institution) to the hierarchy; and fourth, complement the composite total score with component-specific and body-composition outcomes to separate definitional from behavioural pathways and to clarify the divergence between the composite score and the BMI distribution.

## 5 Conclusion

In this large repeated cross-sectional surveillance dataset from one Chinese university, physical-fitness scores showed meaningful clustering within class-year units. Individual BMI, age, sex, and grade, as well as class composition, were associated with the composite score after mutual adjustment, although these associations should not be interpreted causally. Annual composite-score trends were not consistently concordant with the distribution of BMI categories: recorded scores followed a non-monotonic pattern, whereas BMI-defined abnormality increased from 23.3% in 2020 to 31.4% in 2024. This divergence was already evident in 2020–2021 and persisted across the available quality checks and sensitivity analyses. Because BMI is a limited anthropometric screening indicator, the findings do not demonstrate deterioration in overall health; they instead show that a weighted composite score and the BMI distribution provide related but non-interchangeable surveillance information. University fitness analyses should therefore account for class-level clustering and report composite scores alongside BMI categories and component-specific outcomes.

## Data Availability

The datasets analysed for this study are individual-level student physical-fitness surveillance records held by the source institution and are subject to institutional data-protection agreements and the approved ethics protocol; they contain potentially identifying administrative information and therefore cannot be made publicly available. De-identified data may be available from the corresponding author on reasonable request and subject to approval by the data-holding institution and the relevant ethics committee.
