# Supplementary material for: Between-class variation and compositional correlates of physical fitness, and the concordance of composite scores with BMI-defined abnormality: a multilevel analysis of annual surveillance records from one Chinese university
Source: Front Public Health. 2026 Sep 7;14:1896650. doi: 10.3389/fpubh.2026.1896650 (PMC13590708; doi:10.3389/fpubh.2026.1896650)
Supplement: Supplementary file 1 [file Data_sheet_1.docx]

**Supplementary Material**

**Manuscript:** Between-class variation and compositional correlates of physical fitness, and the concordance of composite scores with BMI-defined abnormality: a multilevel analysis of annual surveillance records from one Chinese university.

Tables are presented in landscape orientation with narrow margins so that all columns are shown at full width. Header rows repeat when a table spans a page break.

# **Supplementary Table S1. Selected estimates from single-year multilevel models**

Each model is a random-intercept multilevel model fitted within a single survey year (each student contributes at most one record), with the same individual- and class-level predictors as Model 2 in the main text (calendar-year terms omitted). The table presents the intraclass correlation coefficients (ICCs) and selected key coefficients; other covariates (age, academic grade, class size) were included in each model but are not displayed. Coefficients are unstandardized (B). “BMI (within)” is the individual within-class BMI coefficient; “Prop. male” and “Mean BMI” are class-level coefficients.

| **Year** | **n** | **Units** | **ICC null** | **ICC adj** | **BMI (within)** | **Sex** | **Prop. male** |
| --- | --- | --- | --- | --- | --- | --- | --- |
| 2020 | 17,302 | 452 | 0.097 | 0.093 | −0.455*** | −0.448*** | −0.890 |
| 2021 | 18,195 | 490 | 0.132 | 0.084 | −0.526*** | −4.771*** | 0.096 |
| 2022 | 23,003 | 536 | 0.253 | 0.168 | −0.538*** | −9.003*** | 2.718*** |
| 2023 | 23,484 | 542 | 0.129 | 0.092 | −0.644*** | −4.332*** | 2.027*** |
| 2024 | 24,477 | 571 | 0.117 | 0.080 | −0.461*** | −1.348*** | 0.732 |

***p < 0.001. Mean class BMI coefficients (all negative): 2020 −0.451 (p = 0.014); 2021 −0.553 (p < 0.001); 2022 −1.718 (p < 0.001); 2023 −1.726 (p < 0.001); 2024 −0.291 (p = 0.027). Class-size coefficients were small throughout (range −0.041 to +0.014).

# **Supplementary Table S2. Variance inflation factors (Model 2)**

| **Predictor** | **VIF** | **Predictor** | **VIF** |
| --- | --- | --- | --- |
| Grade 2 | 1.56 | Prop. male (centred) | 1.52 |
| Grade 3 | 1.69 | Mean class BMI (centred) | 1.24 |
| Grade 4 | 1.94 | Year 2021 | 1.80 |
| Sex | 1.44 | Year 2022 | 1.86 |
| BMI (within-class) | 1.03 | Year 2023 | 2.03 |
| Age (centred) | 1.54 | Year 2024 | 1.95 |
| Class size (centred) | 1.07 |  |  |

All VIFs are below the conventional threshold of 5, indicating no problematic multicollinearity.

# **Supplementary Table S3. Additional sensitivity models**

Model 2 is the primary specification. Model 2a adds mean class age as an additional level-2 predictor; Model 2b removes individual age; Model 2c removes academic grade. All models were fitted by maximum likelihood with calendar-year fixed effects; unstandardized coefficients (B) are shown. A dash indicates a predictor not included in that model.

| **Coefficient** | **Model 2** | **2a (+ mean class age)** | **2b (drop age)** | **2c (drop grade)** |
| --- | --- | --- | --- | --- |
| BMI (within-class) | −0.528*** | −0.527*** | −0.528*** | −0.528*** |
| Sex (male vs female) | −4.174*** | −4.145*** | −4.218*** | −4.184*** |
| Class size (centred) | −0.012* | −0.006 | −0.011* | −0.012* |
| Prop. male (centred) | 1.090*** | 0.965*** | 1.117*** | 1.072*** |
| Mean class BMI (centred) | −0.906*** | −0.984*** | −0.921*** | −0.915*** |
| Grade 2 (vs 1) | 0.803*** | 0.223 | 0.697*** | — |
| Grade 4 (vs 1) | 1.913*** | 0.722*** | 1.703*** | — |
| Age (centred) | −0.128*** | −0.203*** | — | −0.087*** |
| Mean class age (centred) | — | 0.760*** | — | — |

*p < 0.05; ***p < 0.001. The class-level associations (proportion male, mean class BMI) and individual-level associations (within-class BMI, sex) are stable across all four specifications. The auxiliary age–grade regression R² = 0.269 (implied VIF = 1.37) confirms low collinearity between age and grade.

# **Supplementary Table S4. Year-specific anthropometric distributions and BMI category prevalence, 2020–2024**

Values are for the retained analytic sample (n = 106,461). Height, weight, and BMI are mean ± SD. BMI categories follow the sex-specific National Student Physical Fitness Standards cut-points (underweight: BMI < 17.2 kg/m² female and < 17.9 kg/m² male; overweight 24 ≤ BMI < 28; obesity BMI ≥ 28). “Abnormal” is the combined prevalence of underweight, overweight, and obesity. Height, weight, and BMI category were complete (0% missing) in every year.

| **Year** | **N** | **Height (cm)** | **Weight (kg)** | **BMI (kg/m²)** | **Underweight %** | **Normal %** | **Overweight %** | **Obese %** | **Abnormal %** |
| --- | --- | --- | --- | --- | --- | --- | --- | --- | --- |
| 2020 | 17,302 | 163.7 ± 8.5 | 57.4 ± 11.2 | 21.32 ± 3.25 | 6.43 | 76.68 | 12.54 | 4.35 | 23.32 |
| 2021 | 18,195 | 163.5 ± 8.4 | 57.5 ± 11.3 | 21.41 ± 3.34 | 6.35 | 75.85 | 13.03 | 4.77 | 24.15 |
| 2022 | 23,003 | 164.9 ± 8.5 | 58.7 ± 11.9 | 21.49 ± 3.43 | 6.42 | 74.57 | 13.90 | 5.11 | 25.43 |
| 2023 | 23,484 | 165.1 ± 8.5 | 60.3 ± 12.3 | 22.03 ± 3.60 | 5.15 | 71.19 | 16.95 | 6.72 | 28.81 |
| 2024 | 24,477 | 164.7 ± 8.4 | 59.5 ± 13.1 | 21.80 ± 3.81 | 8.11 | 68.60 | 16.35 | 6.94 | 31.40 |

Mean BMI rose to 2023 and then fell slightly in 2024, whereas abnormality continued to rise, because the 2024 change was a spreading of the distribution (SD 3.60 → 3.81) with growth in both the underweight and the overweight/obese tails rather than a uniform upward shift.

# **Supplementary Table S5. Data-quality and sensitivity checks for the annual BMI-abnormality trend**

Terminal-digit heaping is the proportion of records whose height (or weight) ended in an integer value; a sharply higher value flags a change in recording or rounding practice. Body weight below 35 kg was treated as a sensitivity-screening threshold rather than as an exclusion criterion or evidence of data error. “Duplicates” are exact duplicate records; “Constant-value units” are class-year units of ≥ 5 records with a single height or weight value. “Standardized abnormality” is direct sex- and grade-standardisation to the pooled composition; “Sensitivity prevalence” repeats the abnormality prevalence after excluding records with weight < 35 kg.

| **Year** | **Height integer, %** | **Weight integer, %** | **Very-low weight (<35 kg), n** | **Duplicates, n** | **Constant-value units, n** | **Standardized abnormality, %** | **Sensitivity prevalence, %** |
| --- | --- | --- | --- | --- | --- | --- | --- |
| 2020 | 10.0 | 10.3 | 8 | 0 | 0 | 23.56 | 23.29 |
| 2021 | 9.9 | 10.2 | 6 | 0 | 0 | 24.67 | 24.12 |
| 2022 | 28.2 | 28.6 | 5 | 0 | 0 | 25.33 | 25.42 |
| 2023 | 10.1 | 10.1 | 4 | 0 | 0 | 28.57 | 28.80 |
| 2024 | 10.0 | 10.0 | 61 | 0 | 0 | 31.07 | 31.23 |

The only year with marked terminal-digit heaping was 2022 (about 28% integer endings), not 2024. No exact duplicate records and no constant-value units were present in any year. Excluding the 61 very-low-weight 2024 records changed the 2024 prevalence only from 31.40% to 31.23%, and sex/grade standardisation preserved the monotonic rise. Half-unit (0.5) endings were not separately computed. Adjusted logistic year contrasts versus 2020 (age, sex, grade; cluster-robust) gave odds ratios of 1.08 (2021), 1.10 (2022), 1.31 (2023), and 1.47 (2024), all p ≤ 0.005 (full estimates in Table S6). Among 368 administrative class codes observed in both the 2023 and 2024 files (matched by class code), 59% showed a higher recorded 2024 abnormality prevalence (mean change +2.9 percentage points), in both sexes and all grades; these comparisons do not represent within-student change because class membership may have differed between years.

# **Supplementary Table S6. Full annual estimates for the composite score and BMI-defined abnormality, 2020–2024**

Adjusted composite-score estimates are estimated marginal means from a linear model with age, sex, and academic grade and class-year cluster-robust standard errors. BMI-abnormality prevalence is directly standardised to the pooled sex and grade distribution; adjusted odds ratios (ORs) versus 2020 are from a logistic model with the same covariates and cluster-robust standard errors. Adjacent-year rows compare each year with the preceding year. All values are shown with 95% confidence intervals (CIs).

(a) Composite fitness score

| **Year** | **Adjusted mean [95% CI]** | **Difference vs 2020 [95% CI]** | **Adjacent-year difference [95% CI]** |
| --- | --- | --- | --- |
| 2020 | 70.68 [70.46, 70.90] | reference | — |
| 2021 | 73.09 [72.82, 73.36] | +2.41 [2.06, 2.76] | +2.41 [2.06, 2.76] |
| 2022 | 69.93 [69.56, 70.30] | -0.75 [-1.18, -0.32] | -3.16 [-3.63, -2.69] |
| 2023 | 71.06 [70.78, 71.34] | +0.38 [0.03, 0.73] | +1.13 [0.67, 1.59] |
| 2024 | 73.23 [72.98, 73.48] | +2.55 [2.21, 2.88] | +2.17 [1.79, 2.54] |

(b) BMI-defined abnormality

| **Year** | **Standardized prevalence, % [95% CI]** | **Adjusted OR vs 2020 [95% CI]** | **p (vs 2020)** |
| --- | --- | --- | --- |
| 2020 | 23.56 [22.93, 24.18] | 1.00 (reference) | — |
| 2021 | 24.67 [24.04, 25.30] | 1.08 [1.02, 1.14] | 0.004 |
| 2022 | 25.33 [24.77, 25.88] | 1.10 [1.05, 1.16] | <0.001 |
| 2023 | 28.57 [28.00, 29.14] | 1.31 [1.24, 1.37] | <0.001 |
| 2024 | 31.07 [30.50, 31.64] | 1.47 [1.40, 1.54] | <0.001 |

Crude (unstandardised) abnormality prevalences were 23.32% (2020), 24.15% (2021), 25.43% (2022), 28.81% (2023), and 31.40% (2024). Adjacent-year adjusted ORs were 1.04 (2020–2021), 1.04 (2021–2022), 1.18 (2022–2023), and 1.13 (2023–2024).

# **Supplementary Table S7. Fixed-effect estimates under the random-intercept (Model 2) and random-slope (Model 3) specifications**

Model 2 is the random-intercept specification reported in the main text (Table 3). Model 3 adds a random slope for within-class BMI across class-year units. The two specifications yield materially identical fixed-effect estimates, confirming that the substantive individual- and class-level conclusions do not depend on the random-effects structure. Model 3 improved overall fit relative to Model 2 (AIC 728,734 vs 729,193; BIC 728,907 vs 729,347; LR χ² = 463.07, df = 2, p < 0.001), and the estimated random BMI-slope variance was 0.073 (SD ≈ 0.27) with a small intercept–slope correlation (0.05).

| **Predictor** | **Model 2: B** | **Model 3: B [95% CI]** |
| --- | --- | --- |
| Grade 2 (vs 1) | 0.803 | 0.78 [0.47, 1.09] |
| Grade 3 (vs 1) | 0.283 | 0.25 [-0.07, 0.57] |
| Grade 4 (vs 1) | 1.913 | 1.86 [1.52, 2.20] |
| Sex (male vs female) | −4.174 | -4.17 [-4.27, -4.06] |
| BMI (within-class) | −0.528 | -0.52 [-0.54, -0.50] |
| Age (centred) | −0.128 | -0.12 [-0.17, -0.08] |
| Class size (centred) | −0.012 | -0.01 [-0.02, -0.00] |
| Prop. male (centred) | 1.090 | 1.08 [0.55, 1.61] |
| Mean class BMI (centred) | −0.906 | -0.87 [-1.05, -0.69] |
| Year 2021 (vs 2020) | 2.805 | 2.83 [2.41, 3.26] |
| Year 2022 (vs 2020) | −0.372 | -0.32 [-0.74, 0.10] |
| Year 2023 (vs 2020) | 1.062 | 1.10 [0.66, 1.53] |
| Year 2024 (vs 2020) | 3.057 | 3.04 [2.62, 3.45] |

Model 2 values are the primary estimates from main-text Table 3. Model 3 values are from the random-intercept-and-slope model. Differences between specifications are negligible for every coefficient (all within rounding of the standardized effect sizes), so the choice between Model 2 and Model 3 does not alter any substantive conclusion.

# **Supplementary Figure S1. Model diagnostics**

**Figure S1.** Left: standardized residuals versus fitted values for Model 2, showing approximately constant variance. Right: normal Q–Q plot of residuals, indicating approximate normality with mild left skew (−0.48), inconsequential at this sample size.

# **Supplementary Figure S2. Annual BMI distribution and category composition**

**Figure S2.** (A) BMI category composition by survey year, showing the growth of the overweight and obese fractions across 2020–2024 and the rise in the underweight fraction in 2024. (B) Kernel density of individual BMI by year; dashed lines mark the overweight (24 kg/m²) and obesity (28 kg/m²) cut-points. The 2024 distribution is broader (higher SD) than earlier years, consistent with growth in both tails rather than a uniform shift.
